# Uremic Toxin Concentrations are Related to Residual Kidney Function in the Pediatric Hemodialysis Population

**DOI:** 10.3390/toxins11040235

**Published:** 2019-04-24

**Authors:** Evelien Snauwaert, Els Holvoet, Wim Van Biesen, Ann Raes, Griet Glorieux, Johan Vande Walle, Sanne Roels, Raymond Vanholder, Varvara Askiti, Karolis Azukaitis, Aysun Bayazit, Nur Canpolat, Michel Fischbach, Nathalie Godefroid, Saoussen Krid, Mieczyslaw Litwin, Lukasz Obrycki, Fabio Paglialonga, Bruno Ranchin, Charlotte Samaille, Franz Schaefer, Claus Peter Schmitt, Brankica Spasojevic, Constantinos J. Stefanidis, Maria Van Dyck, Koen Van Hoeck, Laure Collard, Sunny Eloot, Rukshana Shroff

**Affiliations:** 1 Ghent University Hospital, 9000 Ghent, Belgium; els.holvoet@uzgent.be (E.H.); wim.vanbiesen@ugent.be (W.V.B.); ann.raes@ugent.be (A.R.); griet.glorieux@ugent.be (G.G.); johan.vandewalle@uzgent.be (J.V.W.); Raymond.vanholder@ugent.be (R.V.); sunny.eloot@ugent.be (S.E.); 2 Department of Data Analysis, Faculty of Psychology and Pedagogy, Ghent University, 9000 Ghent, Belgium; sanne.roels@ugent.be; 3 A & P Kyriakou Children’s Hospital, 11527 Athens, Greece; vaskiti@gmail.com (V.A.); cjstefanidis@gmail.com (C.J.S.); 4 Clinic of Pediatrics, Faculty of Medicine, Vilnius University, 08406 Vilnius, Lithuania; k.azukaitis@gmail.com; 5 Department of Pediatric Nephrology, Cukurova University, 01330 Adana, Turkey; ayskar@cu.edu.tr; 6 Department of Pediatric Nephrology, Istanbul University Cerrahpasa School of Medicine, 34096 Istanbul, Turkey; ncanpolat2000@hotmail.com; 7 Children’s Dialysis Center, 67098 Strasbourg, France; fischbam@gmail.com; 8 Department of Pediatric Nephrology, Université Catholique Louvain, 1200 Brussels, Belgium; nathalie.godefroid@uclouvain.be; 9 Hôpital Necker-Enfants Malades, 75015 Paris, France; saoussen.krid@aphp.fr; 10 Department of Nephrology, Kidney Transplantation and Hypertension, Children’s Memorial Health Institute, 04-730 Warsaw, Poland; M.Litwin@IPCZD.PL (M.L.); obrycki.lukasz@gmail.com (L.O.); 11 Pediatric Nephrology Dialysis and Transplant Unit, Fondazione IRCCS Ca’ Granda Ospedale Maggiore Policlinico, 20122 Milan, Italy; fabio.paglialonga@policlinico.mi.it; 12 Hôpital Femme Mère Enfant, Hospices Civils de Lyon, 69677 Bron, France; bruno.ranchin@chu-lyon.fr; 13 CHU Lille, Service de Néphrologie Pédiatrique, 59000 Lille, France; charlotte.samaille@chru-lille.fr; 14 Center for Pediatrics and Adolescent Medicine, 69120 Heidelberg, Germany; franz.schaefer@med.uni-heidelberg.de (F.S.); clauspeter.schmitt@med.uni-heidelberg.de (C.P.S.); 15 University Children’s Hospital, Belgrade and School of Medicine, University of Belgrade, 11000 Belgrade, Serbia; brankicaspasojevic@yahoo.com; 16 Department of Pediatric Nephrology, Leuven University Hospital, 3000 Leuven, Belgium; maria.vandyck@uzleuven.be; 17 Department of Pediatric Nephrology, Antwerp University, 2650 Antwerp, Belgium; koen.vanhoeck@uza.be; 18 Department of Pediatric Nephrology, CHU Liège, 4000 Liège, Belgium; laure.collard@chc.be; 19 Great Ormond Street Hospital for Children NHS Foundation Trust, London WC1N 3JH, UK; rukshana.shroff@gosh.nhs.uk

**Keywords:** chronic kidney disease, end-stage kidney disease, child, uremic toxins, hemodialysis, residual kidney function

## Abstract

Protein-bound uremic toxins (PBUTs) play a role in the multisystem disease that children on hemodialysis (HD) are facing, but little is known about their levels and protein binding (%PB). In this study, we evaluated the levels and %PB of six PBUTs cross-sectionally in a large pediatric HD cohort (*n* = 170) by comparing these with healthy and non-dialysis chronic kidney disease (CKD) stage 4–5 (*n* = 24) children. In parallel β2-microglobulin (β2M) and uric acid (UA) were evaluated. We then explored the impact of age and residual kidney function on uremic toxin levels and %PB using analysis of covariance and Spearman correlation coefficients (*r_s_*). We found higher levels of β2M, p-cresyl glucuronide (pCG), hippuric acid (HA), indole acetic acid (IAA), and indoxyl sulfate (IxS) in the HD compared to the CKD4–5 group. In the HD group, a positive correlation between age and pCG, HA, IxS, and pCS levels was shown. Residual urine volume was negatively correlated with levels of β2M, pCG, HA, IAA, IxS, and CMPF (*r_s_* −0.2 to −0.5). In addition, we found overall lower %PB of PBUTs in HD versus the CKD4–5 group, and showed an age-dependent increase in %PB of IAA, IxS, and pCS. Furhtermore, residual kidney function was overall positively correlated with %PB of PBUTs. In conclusion, residual kidney function and age contribute to PBUT levels and %PB in the pediatric HD population.

## 1. Introduction

Children in end-stage kidney disease (ESKD) are facing a multisystem disease with lifelong consequences, such as frequent hospitalizations, decreased quality of life, and a short expected lifetime compared to healthy children [1,2,3]. Several factors contribute to the systemic nature and the accompanying high mortality and morbidity faced by children in ESKD: e.g., deterioration of renal endocrine and homeostatic function, problems and/or consequences related to kidney disease and its treatment, and the accumulation of toxic organic metabolites (i.e., uremic toxins). Based on studies in the adult ESKD population, the accumulation of uremic toxins is considered a major determinant in the pathophysiology of ESKD, nevertheless, no studies in the pediatric population have been performed [2,4,5,6]. To date, more than 150 uremic solutes have been described, which can be divided into three categories, based on their physicochemical characteristics explaining their behavior during dialysis: small, water-soluble compounds; larger, middle molecules; and protein-bound toxins (PBUTs) [7].

PBUTs have the same molecular weight as small water-soluble uremic compounds, however, PBUTs’ accumulation and removal are far more complex [8]. Due to their protein binding (PB), only the free fraction of PBUTs can diffuse to the dialysate, and consequently their removal is strongly limited during dialysis [8]. Furthermore, both albumin levels and binding affinity are decreased in ESKD, which is also considered to impact %PB, and thus, the accumulation and removal of PBUTs [9,10]. Studies in adult ESKD patients have confirmed that plasma levels of PBUTs are poorly controlled by current hemodialysis (HD) strategies, including hemodiafiltration and extended nocturnal hemodialysis [11,12,13,14]. Instead, levels of PBUTs in adult patients on maintenance HD were found to be very effectively eliminated by residual kidney function [15]. In addition to greater removal of these compounds, residual kidney function has also been associated with beneficial effects on pediatric and adult patient outcomes: e.g., improved albumin binding affinity, less inflammation; better growth; improved volume, mineral and electrolyte control; less myocardial stunning; decreased mortality; and most importantly a better quality of life [16,17,18,19,20,21,22]. The association between growth and residual kidney function is especially relevant for the pediatric population, since growth is an important clinical outcome due to its association with quality of life, risk of hospitalization, and death [23,24].

To date, PBUT levels and %PB, and their relation to residual kidney function, remain unexplored in the pediatric HD population. Nevertheless, substantial differences can be expected compared to adults, since children have lower circulating proteins, higher protein and caloric needs per kilogram body weight, a different pattern of underlying kidney disease, maturational changes in organic solute transport at the proximal tubule, and an ongoing development of intestinal microbiota [25,26,27,28]. Therefore, we first described PBUT levels and %PB in children on maintenance HD by comparing these values with those of non-dialyzed chronic kidney disease stage 4–5 children (CKD4–5), and by correlating these values with age. Second, we explored the relation between PBUT levels, %PB, and residual kidney function, by comparing anuric and non-anuric children on HD, and by evaluating their association with residual urine volume. In parallel, the small water-soluble compound uric acid (UA) and the middle molecule β2-microglobullin (β2M) were evaluated.

## 2. Results

### 2.1. Patient and Treatment Characteristics

**Figure A1:**
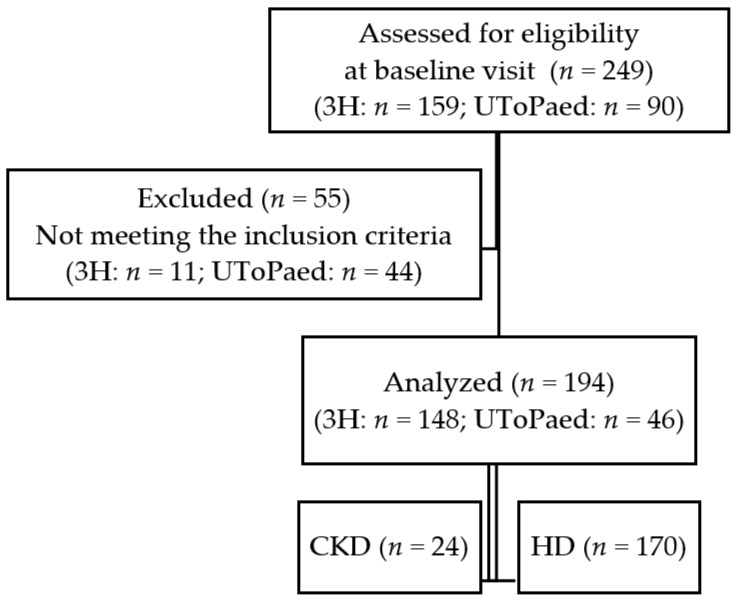
Flow diagram.

Of the 249 children from both studies assessed for eligibility, 194 children were included and analyzed in this study (Figure A1). Twenty-four children with CKD4–5, and 170 children on maintenance HD (101 non-anuric and 69 anuric) were included in the study. The general and treatment specific characteristics of the included children according to their residual kidney function are presented in Table 1. Children with CKD4–5 had a median estimated glomerular filtration rate (eGFR) of 17 [11; 23] mL/min/1.73 m^2^. Gender distribution was equal across groups. The distribution of age and primary kidney diseases were different between groups (*p* < 0.01 and *p* = 0.03, respectively). Congenital anomalies of kidney and urinary tract (CAKUT) were most frequently reported in the CKD4–5 (62.5%), and non-anuric HD group (50.5%). In contrast, other or not-specified kidney diseases (42.0%) were most frequent in the anuric HD group. There were no differences between vascular access and dialysis prescriptions between the non-anuric and anuric HD group. The majority of children in both HD group were treated with conventional HD (±70%) with high-flux dialyzers (52.2–60.4%) on a frequency of 3 sessions per week (>93%). The median residual urine volume of the non-anuric HD group was 411 [151; 864] mL/24 h/m^2^. The dialysis vintage was longer in the anuric versus non-anuric HD group, e.g., 1.26 [0.33; 3.65] versus 0.40 [0.14; 1.14] years.

### 2.2. Uremic Toxin Plasma Levels and % Protein Binding in CKD 4–5 versus HD

Total levels and %PB of the evaluated uremic toxins in the CKD4–5 versus HD group are summarized in Table 2. Compared to our previously published cohort of healthy children [29], we found total levels of 7.8-fold higher for indole acetic acid (IAA), up to 43-fold higher for hippuric acid (HA) in children on HD.

Levels of β2M, and total levels of p-cresyl glucuronide (pCG), HA, IAA, and indoxyl sulfate (IxS) were overall lower in the CKD4–5 versus HD group (all *p* ≤ 0.01). The same results were obtained for free levels of PBUTs between CKD4–5 and HD as for total levels of PBUTs (data not shown). No differences in concentration of UA, and total levels of p-cresyl sulfate (pCS) and 3-carboxy-4-methyl-5-propyl-furanpropionic acid (CMPF) between the CKD4-5 and HD group were found. The degree of protein-binding of all evaluated PBUTs was lower in the HD versus CKD4–5 group (all *p* < 0.05, Table 2).

### 2.3. Effect of Age on Uremic Toxin Levels and % Protein-binding in Children on HD

The effect of age on uremic toxin levels and %PB in children on maintenance HD is displayed in Table 3. Irrespectively residual kidney function, increasing age was associated with higher total levels of pCG, HA, IxS and pCS (all *p* < 0.05). In addition, higher %PB was demonstrated for IAA, IxS, and pCS with increasing age (all *p* < 0.05). Levels of CMPF, UA, and β2M were not influenced by age.

### 2.4. Uremic Toxin Plasma Levels and % Protein-Binding in the Anuric versus Non-Anuric HD

**Figure 1:**
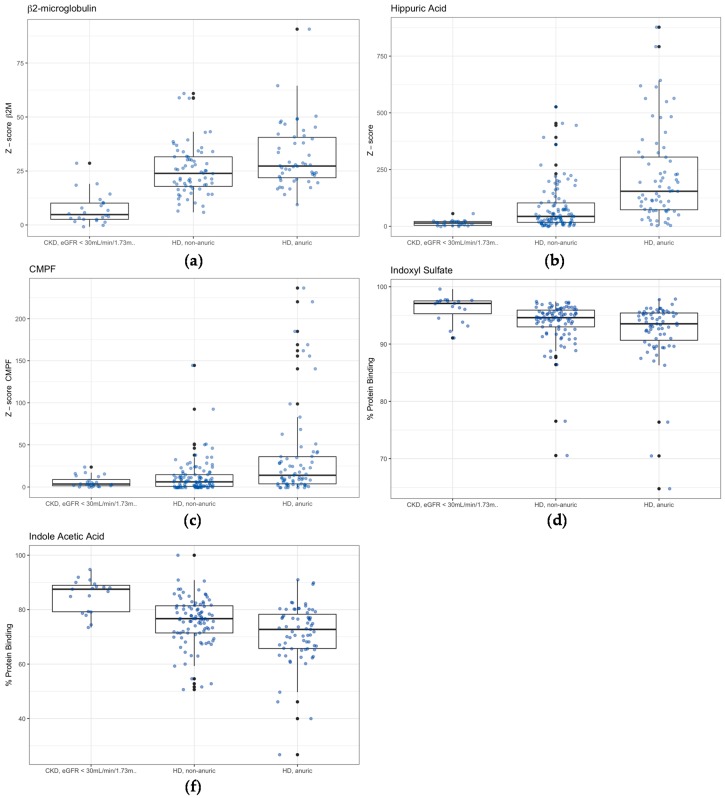
Only significant differences between anuric versus non-anuric hemodialysis (HD) are shown: (**a**) z-score of β2-microglobulin levels (β2M); (**b**) z-score of hippuric acid (HA) total levels; (**c**) z-score of 3-carboxy-4-methyl-5-propyl-furanpropionic acid (CMPF) total levels; (**d**) % protein binding of indoxyl sulfate (IxS); and (**e**) % protein binding of indole acetic acid (IAA). Concentrations of uremic toxin were expressed as a z-score (for child i the $z_{i}=\left(x_{i}-\bar{x_{c}}\right)/sd_{x_{c}}$; with $x_{i}$: concentration of toxin in child i; $\bar{x_{c}}$: average toxin level in the control group; and $sd_{x_{c}}$: standard deviation of uremic toxin in the control group), using control group as described in Snauwaert et al [29]. CKD: chronic kidney disease, eGFR: estimated glomerular filtration rate.

As displayed in Table 4, higher uremic toxin levels in the anuric and non-anuric HD group were found for β2M, HA and CMPF (Figure 1a–c; all *p* ≤ 0.01). %PB in the anuric HD group was lower for HA, IAA, and IxS compared to the non-anuric HD group (Figure 1d,e; all *p* < 0.05).

### 2.5. Uremic Toxin Plasma Levels and % Protein-Binding versus Residual Urine Volume in the HD Group

In Table 5, Spearman’s rho correlation coefficients (*r_s_*) between uremic toxins and residual urine volume (mL/24 h/m^2^) are summarized. Significant *r_s_* values were found between residual urine volume and total levels of β2M, pCG, HA, IAA, IxS, and CMPF (all *p* < 0.01). The highest *r_s_* were found for HA *r_s_* = −0.481) and IxS (*r_s_* = −0.356). Residual urine volume was not correlated with for UA and pCS levels. The same results were obtained for free levels of PBUTs between CKD4–5 and HD as for total levels of PBUTs (data not shown). For all evaluated PBUTs, residual urine volume was positive correlated with %PB and (all *p* ≤ 0.02), of which the highest *r_s_* were found for IAA (*r_s_* = 0.328) and HA (*r_s_* = 0.313).

## 3. Discussion

This is the first detailed description of uremic toxin levels and %PB in children on maintenance HD. The most striking findings are: (1) the substantially higher levels of uremic toxins, and the lower %PB of PBUTs in children on maintenance HD compared to healthy and CKD4–5 children; (2) the positive correlation between age and PBUT levels and their %PB; and (3) the major contribution of residual kidney function on uremic toxin levels and %PB.

First, we described the levels of eight uremic toxins with different physicochemical characteristics affecting their behavior during HD: one small water-soluble toxin (UA), one middle molecule (β2M), and six protein-bound uremic toxins covering a wide range of protein binding (pCG, HA, IAA, IxS, pCS, and CMPF). This is the first study to evaluate a large pediatric cohort during maintenance HD including a broad selection of uremic toxins. The median pre-dialysis levels in children on maintenance HD were overall substantially higher than the levels described in healthy children, as previously described by Snauwaert et al [29]. The total levels of HA increased up to 43-fold relative to normal levels, which is consistent with previous findings in adults [30,31,32,33]. The median total levels of the evaluated uremic toxins in children on maintenance HD were in the same range as in adult uremic patients [31]. We also found that the total levels of β2M, pCG, HA, IAA, and IxS were higher in the HD compared to CKD4–5 group, while the levels of UA, pCS, and CMPF were in the same range. Similarly, the study of Liabeuf et al. [34], evaluating adult patients with CKD, did not find different total pCS levels in CKD5 versus CKD5D (on dialysis). Therefore, pCS levels seem to be poorly related to the remaining kidney function, which dovetails nicely with our previous finding that pCS is not related to eGFR, and the present finding that pCS is not related to residual urine volume [35]. Of note, the variability of uremic toxin levels among individuals was large, especially for the PBUTs, and is in line with the previously described high inter-patient variability of PBUT levels in adult HD patients by Eloot et al. [36].

In addition, we found that the %PB was overall lower in the HD compared to CKD4-5 group, i.e., the free levels of these PBUTs rather than their total levels was higher relative to normal levels. This finding reflects alterations in the binding affinity of albumin, the main transporter of PBUTs, in children on maintenance HD [22,32]. Several factors have been reported to alter %PB of PBUTs during CKD: i.e., lower albumin levels due to increased protein catabolism rate and/or albumin losses, and a lower binding affinity to albumin due to post-translational modifications (e.g., oxidation, carbamylation, nitrosylation, glycation, and acetylation) [9,10,37,38,39]. High total PBUT levels with concomitant competition between different compounds (directly or by induction of allosteric alterations) has also been suggested, but was not confirmed in recent studies [9,40]. This altered PB might impact on overall outcomes of patients with CKD, since an improved %PB enhances the renal clearance of PBUTs by providing a readily accessible reservoir for efficient removal of toxins throughout their passage within the native kidney [32]. In addition, post-translational modifications of albumin have been inversely correlated with clinical outcomes [41,42]. Based on the ‘free drug hypothesis’ that assumes that free drug levels correlates best with drug responses, it could be hypothesized that lower %PB and thus higher free levels of PBUTs, results in increased toxicity [43]. On the contrary, less %PB might also increase the possibility for diffusive dialyzer clearance during maintenance HD, and subsequently facilitate removal of PBUTs.

Second, we found that total levels of pCG, HA, IxS, and pCS were positively related with age, which is also previously demonstrated in the adult CKD population [44]. Remarkably, while age-dependency of IxS and pCS was also found in healthy adults, no positive correlation between age and PBUTs in healthy children was found, suggesting a different basis of age-dependency of PBUTs in adults versus children [45,46]. In addition, we found that, irrespective the albumin levels, %PB of IAA, IxS, and pCS positively correlated with age, whereas especially the youngest children had the lowest %PB, and thus the highest free fraction. How age is influencing PBUT levels and %PB might be related to e.g., a different pattern of underlying kidney disease, and to developmental and nutritional changes throughout childhood, but remians unexplored.

Third, the present study found that residual kidney function substantially contributes to uremic toxins levels in children on maintenance HD. We demonstrated that levels of β2M, HA and CMPF were higher in anuric versus non-anuric children on maintenance HD. In addition, we found a negative correlation between total levels of β2M, pCG, HA, IAA, IxS, and CMPF and residual urine volume. These findings are in agreement with lower plasma levels of middle molecules (e.g., β2M, cystatin C, FGF-23, and β-trace proteins) and PBUTs (i.e., HA, IxS) reported in adult HD/PD patients with residual kidney function compared to those without [15,29,47,48,49,50,51,52]. Due to a preserved active tubular secretion and the continuous clearance capacity of the residual kidney, even a small amount of residual kidney function is accepted to provide a major portion of the total solute removal [15]. In contrast to the studies of Marquez et al. [15] and Eloot et al. [52], we could not demonstrate an impact of residual kidney function on total concentration of pCS. On the other hand, our findings were similar to those of Viaene et al. [50]. The importance of residual kidney function in limiting the accumulation of these PBUTs is further highlighted by the recent study by Leong et al. [53]. The authors demonstrated that patients with residual kidney function on twice weekly hemodialysis had similar total levels of IxS and pCS and even lower levels of HA compared to anuric patients on thrice weekly hemodialysis [53]. In addition, we found a positive correlation between residual urine volume and the %PB for all evaluated PBUTs, e.g., %PB of HA, IAA and IxS, was lower in anuric compared to non-anuric children on HD. The association of altered PB with residual kidney function has been previously described by Klammt et al. [22], who demonstrated higher albumin binding affinity in adults on HD with a preserved urine output of >500 mL/day compared to anuric patients. How preserved residual kidney function improves albumin binding affinity needs further elucidation, and might be related to lower total levels of protein-bound uremic toxins, and/or less post-translational modifications of albumin.

Taking into account the beneficial effect of residual kidney function on (1) overall uremic toxin levels and PB highlighted in this study, and on (2) several patient outcomes (e.g., less inflammation, better growth, improved control of volume and minerals and electrolytes, less myocardial stunning, decreased mortality, and a better quality of life), residual kidney function should be recognized as an important clinical parameter in the management and treatment of children on maintenance HD, while its prevention is an important therapeutic target [16,17,18,19,20,21]. Hence, strategies to preserve residual kidney function need to be incorporated in the clinical management of children on maintenance HD (e.g., minimizing nephrotoxic events, control of blood pressure, and avoidance of intradialytic hypotension) and should be explored in future research [54].

However, one should recognize that measuring residual kidney function by calculating renal urea/creatinine clearances poses several practical hurdles in young children, since bladder catheterization in not-potty trained children is needed to obtain a timed urine collection. In contrast, residual urine volume can be measured more easily and non-invasively using the weight of the wet nappies in not-potty trained children, which might therefore be a valuable alternative for residual renal urea/creatinine clearances, and an indicator of a broad array of uremic toxin levels and %PB as demonstrated in this study. Future studies with longitudinal design will be needed to confirm the importance of residual urine volume in the management of children on maintenance HD.

This study has some weaknesses that should be addressed in future research. First, we did not assess clinical outcomes and therefore cannot specify to what extent high uremic toxin levels and low %PB correlate with clinical outcomes. However, this is a first explorative study in children and we will address their relation with clinical outcomes (e.g., cardiovascular measures, and growth) in future studies. Second, due to the cross-sectional design of the study, we could not adjust for intra-patient variability, which might have decreased our capability to find associations, and should therefore, be addressed in a longitudinal follow-up study. Third, we only registered residual urine volume in this study, and did not evaluate its relation to renal clearances of uremic toxins. Fourth, we have not evaluated uremic toxin levels in children on extended HD or peritoneal dialysis. Finally, we have to recognize that anuric and non-anuric children on maintenance HD were different with respect to underlying kidney disease and dialysis vintage, which might carry a risk of bias. However, this study also has a number of strengths. This study is the first to evaluate uremic toxins with different physicochemical characteristics and variable degree of PB in a large pediatric HD cohort. Children are uniquely suited for this analysis, because the majority of children have an isolated kidney and urinary tract disease, without pre-existing confounding factors (e.g., smoking, ageing) or co-morbidities such as diabetes mellitus, which all were previously identified described to alter uremic toxin levels [46].

## 4. Conclusions

In conclusion, the present study highlighted the importance of residual kidney function, and suggested that residual urine volume might be a valuable indicator of overall uremic toxins levels and %PB in children on maintenance HD. In addition, we found that PBUT levels and %PB are related to age, which should be further elucidated in future research.

## 5. Materials and Methods

### 5.1. Patients and Study Design

Inclusion criteria for the study were either (1) children with CKD with an eGFR <30 mL/min/1.73 m^2^, determined by the updated Schwartz equation, but not on dialysis or (2) children on maintenance hemodialysis (HD) defined as daytime intermittent HD or HDF with individual hemodialysis sessions of maximum 4 h and a total hemodialysis treatment of maximum 16 h per week [55,56]. Children less than one week on dialysis were excluded from the analysis. Furthermore, children with active bacterial or viral infectious disease with implications on the child’s wellbeing, and active systemic inflammatory disease or active malignancy were excluded. The children were recruited between 2011 and 2017 from two prospective multicentric cohorts, the 3H (HDF, heart, height) study and the UToPaed (Uremic Toxins in Paediatrics) study. Both studies were performed according to the principles of the declaration of Helsinki and were approved by the institutional review boards at each participating institution. Written informed consent was obtained from all parents and patients as appropriate (code B670201524922; approval date 05/06/2015). The 3H study is a non-randomized parallel-arm intervention trial performed within the International Pediatric Hemodialysis Network, of which the study protocol has been described previously [57]. The UToPaed study is a Belgian prospective observational study analyzing representative uremic toxins in children (0–18 years) with CKD stage 1 to 5D with the aim to evaluate the relationship of these uremic toxins to cardiovascular risk profile, growth, quality of life, and sleep pattern in the pediatric population.

### 5.2. Residual Kidney Function

The children included in this study were categorized into three groups according to their residual kidney function: (1) anuric HD, (2) non-anuric HD, and (3) non-dialysis CKD with an eGFR <30 mL/min/1.73 m^2^. In children on maintenance HD, residual urine volume was also measured during a 24 h interval and corrected for body surface area, expressed as volume per 24 h per body surface area (mL/24 h/m^2^) and referred to in this manuscript as residual urine volume.

### 5.3. Laboratory Analyses

Blood samples were collected during a routine ambulatory visit in the non-dialyzed CKD group, and before a midweek hemodialysis session in the HD groups. Subsequently, the samples were immediately centrifuged, aliquoted, and stored at −80 °C until batch analysis. Standard laboratory methods were used to measure serum protein and albumin levels. UA and total levels of PBUTs (pCG, HA, IAA, IxS, pCS, and CMPF) were determined in plasma samples which first had been diluted, followed by a protein denaturation step [29]. Subsequently, the samples were centrifuged and filtered through Amicon Ultra 0.5 mL Filters (molecular weight cut-off 30 kDa, Merck KGaA, Darmstadt, Germany). To measure the free fraction, untreated plasma samples were filtered first through the Amicon Ultra Filters. Subsequently, reversed-phase ultra-high-performance liquid chromatography (UPLC) by an Agilent 1290 Infinity device (Agilent, Santa Clara, CA, USA) was performed to analyze the total and free levels of protein-bound uremic toxins. HA and CMPF were assessed with an Agilent G4212A diode array detector at 245 nm, and 254 nm, respectively. IxS (*λ*_ex_: 280 nm, *λ*_em_: 376 nm), pCS and pCG (*λ*_ex_: 264 nm, *λ*_em_: 290 nm), and IAA (*λ*_ex_: 280 nm, *λ*_em_: 350 nm) were assessed by an Agilent G1316C fluorescence detector. Free and total levels of PBUTs were used to calculate their %PB according to the equation:

%PB = ([*T*] − [*F*])/[T] × 100% (1)

where [*T*]: total concentration (mg/dL) and [*F*]: free concentration (mg/dL).

Plasma levels of β2M were determined with a sandwich ELISA from ORGENTEC Diagnostika GmbH (Germany) according to the manufacturer’s guidelines, and analyzed using the EL808 Ultra Microplate Reader from Bio-Tek Instruments (Winooski, VT, USA) using the KC4V3.0 Analysis Software (Bio-Tek^®^Instruments, Winooski, VT, USA).

### 5.4. Statistical Analyses

Continuous variables were shown as median value with [25th; 75th percentile] or mean ± standard deviation, as appropriate. Categorical variables were expressed as frequencies and percentages. Comparison of patient characteristics between the three defined groups (anuric HD, non-anuric HD, and non-dialyzed CKD) were performed using the Chi-square for categorical data and Kruskall-Wallis test for continuous data. For comparison of treatment characteristics between anuric and non-anuric HD group, the Chi-square test was used for categorical data and Mann-Whitney U test for continuous data. To evaluate whether uremic toxin levels and %PB (if applicable) were different between (1) the CKD versus HD group, and (2) the anuric versus non-anuric HD group, analysis of covariance (ANCOVA) was used with statistical control for age, and for the %PB analysis also adjustment for serum albumin was added. Dunnett’s test correction for multiple testing was applied per test and when all uremic toxins were mutually compared [58]. ANCOVA was also used for the analysis of age on uremic toxin levels, with adjustment for residual kidney function and %PB (solely in protein binding analysis). Spearman’s rho (*r_s_*) correlation coefficients were calculated to correlate uremic toxin levels and %PB (if applicable) with residual urine volume (mL/24 h/m^2^). Given the low percentage of missing data (<1%), the data was considered missing at random. A *p*-value < 0.05 was considered statistically significant. All statistical analyses were performed using *R* version 3.3.1 (*R*-Core Team, 2016) [59].

## Figures and Tables

**Table 1:** General patient characteristics.

	CKD <30 mL/min/1.73 m^2^	Non-Anuric HD	Anuric HD	*p*
Number	24	101	69	
Age (years)	8.4 [3.7; 14.8]	13.5 [9.6; 15.8]	15.1 [11.7; 16.9]	<0.01
Male (%)	17 (70.8)	56 (55.4)	35 (50.7)	0.23
**Kidney disease (%)**				
Glomerular	4 (16.7)	18 (17.8)	17 (24.6)	0.03
CAKUT	15 (62.5)	51 (50.5)	21 (30.4)	
Cystic disease	1 (4.2)	3 (3.0)	2 (2.9)	
Other or unknown	4 (16.7)	29 (28.7)	29 (42.0)	
**eGFR (mL/min/1.73 m^2^)**	17 [11; 23]	-	-	-
**Vascular access (%)**				
AVG/AVF	-	35 (34.7)	30 (43.5)	0.39
CVC	-	66 (65.3)	39 (56.5)	
**Dialysis modality**				
HDF (%)	-	31 (30.7)	22 (31.9)	0.87
Conventional HD (%)	-	70 (69.3)	47 (68.1)	
**Dialyzed blood volume**				
Blood flow (mL/BSA)	-	176 [141; 214]	180 [141; 213]	0.78
Dialysis hours/week (h)	-	10.5 ± 1.2	10.7 ± 1.2	0.33
**Dialyzer**				
Low-flux (%) ^$^	-	40 (39.6)	33 (47.8)	0.34
High-flux (%)	-	61 (60.4)	36 (52.2)	
**Dialysis sessions/week (%)**				
Three	-	94 (93.1)	66 (95.7)	0.77
Two or four	-	7 (6.9)	3 (4.3)	
**Average ultrafiltration (mL/m^2^)**	-	968 [364; 1334]	1364 [755; 1675]	<0.01
**Residual kidney function**				
Volume (mL/24 h/m^2^)	-	411 [151; 864]	-	-
<100 mL/24 h	-	9 (8.9)	-	-
100–200 mL/24 h	-	14 (13.9)	-	-
200–500 mL/24 h	-	24 (23.8)	-	-
+500 mL/24 h	-	54 (53.5)	-	-
**Dialysis vintage (years)**	-	0.40 [0.14; 1.14]	1.26 [0.33; 3.65]	<0.01
**Routine blood results**				
Albumin (g/L)	43 [41; 47]	40 [38; 43]	41 [38; 43]	<0.01

Data are median [25th; 75th percentile] or frequency (percentage), as appropriate. *p*-values of comparison of patient characteristics between the 2 or 3 groups (as appropriate). ^$^ Low-flux dialyzer is defined by a dialyzer membrane ultrafiltration coefficient (KUF) of <20 mL/h/mmHg. CKD: chronic kidney disease, eGFR: estimated glomerular filtration rate, HD: hemodialysis, BSA: body surface area, HDF: hemodiafiltration, CAKUT: congenital anomaly of kidney and urinary tract; AVF: arteriovenous fistula; AVG: arteriovenous graft; CVC: central venous catheter; RKD: residual kidney diuresis (mL/24 h/m^2^).

**Table 2:** Plasma levels and protein binding of uremic toxins in children with non-dialysis CKD stage 4–5 compared to children on maintenance HD (hemodialysis).

	Healthy Children [29]	CKD < 30 mL/min/1.73 m^2^ (*n* = 24)	Maintenance HD (*n* = 170)	Ratio HD/Healthy	*p* *
**Water-soluble uremic toxins**					
Uric acid (mg/dL)	-	7.36 [6.55; 8.93]	7.02 [5.80; 8.06]	-	0.09
**Middle molecules**					
β2-microglobulin (µg/mL)	1.74 ± 0.34	9.92 [7.72; 15.4]	30.7 [4.4; 39.3]	17.6	**<0.01**
**Protein-bound uremic toxins**					
p-cresyl glucuronide					
Total levels (mg/dL)	0.01 ± 0.01	0.02 [0.01; 0.06]	0.15 [0.03; 0.33]	25.0	**<0.01**
Protein binding (%)	17 [0; 31]	18.6 [9.18; 23.1]	9.31 [5.24; 12.7]	-	**<0.01**
Hippuric acid					
Total levels (mg/dL)	0.04 ± 0.04	0.39 [0.13; 0.64]	1.89 [0.72; 3.43]	43.0	**<0.01**
Protein binding (%)	64 [53; 70]	62.8 [55.1; 66.4]	51.7 [45.7; 58.5]	-	**<0.01**
Indole acetic acid					
Total levels (mg/dL)	0.02 ± 0.01	0.06 [0.04; 0.08]	0.18 [0.12; 0.27]	7.8	**<0.01**
Protein binding (%)	90 [88; 94]	87.1 [79.2; 89.2]	76.1 [69.2; 80.2]	-	**<0.01**
Indoxyl sulfate					
Total levels (mg/dL)	0.06 ± 0.03	0.56 [0.44; 0.73]	2.04 [1.37; 2.70]	36.4	**<0.01**
Protein binding (%)	94 [89; 99]	97.0 [94.9; 97.6]	94.5 [91.9; 95.6]	-	**0.02**
p-cresyl sulfate					
Total levels (mg/dL)	0.24 ± 0.18	1.67 [0.93; 2.31]	2.35 [1.03; 3.27]	9.6	0.82
Protein binding (%)	95 [91; 98]	97.4 [95.4; 97.7]	92.4 [88.4; 94.8]	-	**0.03**
CMPF					
Total levels (mg/dL)	0.01 ± 0.01	0.05 [0.02; 0.21]	0.12 [0.03; 0.33]	12.0	0.97

Data are median [25th; 75th percentile], *p* *: compares uremic toxins in children on maintenance HD versus children with CKD stage 4–5 (eGFR < 30 mL/min.1.73 m^2^). Significant *p*-values are marked in bold. CKD: chronic kidney disease, HD: hemodialysis.

**Table 3:** Association of age and uremic toxin levels and %PB in children on maintenance HD.

AgeΔ (*n* = 170)	Estimate (SE)	*p* ^µ^
**Small water-soluble uremic toxins**		
Uric Acid	0.00 (0.00)	0.48
**Middle molecules**		
β2-microglobulin	0.00 (0.00)	0.56
**Protein-bound uremic toxins**		
p-cresyl glucuronide		
Total levels (mg/dL)	0.03 (0.01)	**0.03**
Protein binding (%)	−0.08 (0.16)	0.93
Hippuric acid		
Total levels (mg/dL)	0.03 (0.01)	**0.01**
Protein binding (%)	−0.04 (0.22)	0.99
Indole acetic acid		
Total levels (mg/dL)	−0.001 (0.006)	0.99
Protein binding (%)	0.54 (0.19)	**0.01**
Indoxyl sulfate		
Total levels (mg/dL)	0.01 (0.01)	**0.03**
Protein binding (%)	0.21 (0.09)	**0.03**
p-cresyl sulfate		
Total levels (mg/dL)	0.03 (0.01)	**<0.01**
Protein binding (%)	0.74 (0.23)	**<0.01**
CMPF		
Total levels (mg/dL)	0.04 (0.02)	0.08

Data are (log transformed) estimates with (standard error). *p* ^µ^: association of uremic toxin concentration and age (years). ^Δ^ Analysis corrected for residual kidney function. Significant p-values are marked in bold. SE: standard error.

**Table 4:** Plasma levels and %PB of uremic toxins between anuric and non-anuric children on maintenance HD.

Residual Kidney Function in HD	Non-Anuric HD ^$^ (*n* = 101)	Anuric HD ^$^ (*n* = 69)	*p* *
**Small water-soluble uremic toxins**			
UA (mg/dL)	6.75 [5.78; 7.70]	7.44 [6.06; 8.33]	0.31
**Middle molecules**			
β2M (µg/mL)	29.0 [22.7; 36.8]	32.4 [26.7; 46.0]	**0.01**
**Protein-bound uremic toxins**			
p-cresyl glucuronide			
Total levels (mg/dL)	0.11 [0.03; 0.30]	0.19 [0.07; 0.42]	0.08
Protein binding (%)	9.58 [6.46; 13.6]	7.83 [3.36; 12.3]	0.56
Hippuric acid			
Total levels (mg/dL)	1.13 [0.57; 2.63]	3.27 [1.84; 5.56]	**<0.01**
Protein binding (%)	54.1 [48.9; 60.1]	49.2 [44.0; 54.8]	0.04
Indole acetic acid			
Total levels (mg/dL)	0.16 [0.11; 0.25]	0.20 [0.14; 0.28]	0.44
Protein binding (%)	76.5 [71.7; 81.4]	72.7 [65.7; 78.3]	**<0.01**
Indoxyl sulfate			
Total levels (mg/dL)	1.89 [1.09; 2.48]	2.29 [1.77; 3.02]	0.06
Protein binding (%)	94.7 [93.2; 96.7]	93.4 [90.4; 95.4]	**<0.01**
p-cresyl sulfate			
Total levels (mg/dL)	2.32 [0.92; 3.09]	2.50 [1.05; 3.80]	0.89
Protein binding (%)	93.5 [88.9; 95.4]	91.3 [87.4; 94.0]	0.12
CMPF			
Total levels (mg/dL)	0.08 [0.02; 0.19]	0.18 [0.05; 0.44]	**<0.01**

Data are median [25th; 75th percentile]. *p* *: compares uremic toxins in anuric HD children versus non-anuric HD children. ^$^ Analysis corrected for age. Significant p-values are marked in bold. HD: hemodialysis.

**Table 5:** Spearman’s rho (*r_s_*) correlation coefficients between uremic toxins and residual urine volume (mL/24 h/m^2^) in children on maintenance HD.

	*r_s_*	*p* *
**Small water-soluble uremic toxins**		
Uric Acid (mg/dL)	−0.144	0.06
**Middle molecules**		
β2-microglobulin (µg/mL)	−0.279	**<0.01**
**Protein-bound uremic toxins**		
p-cresyl glucuronide		
Total levels (mg/dL)	−0.259	**<0.01**
Protein binding (%)	0.182	**0.02**
Hippuric acid		
Total levels (mg/dL)	−0.481	**<0.01**
Protein binding (%)	0.313	**<0.01**
Indole acetic acid		
Total levels (mg/dL)	−0.229	**<0.01**
Protein binding (%)	0.328	**<0.01**
Indoxyl sulfate		
Total levels (mg/dL)	−0.356	**<0.01**
Protein binding (%)	0.252	**<0.01**
p-cresyl sulfate		
Total levels (mg/dL)	−0.129	0.10
Protein binding (%)	0.191	**0.01**
CMPF		
Total levels (mg/dL)	−0.218	**<0.01**

*r_s_*: Spearman’s rho correlation coefficients. *p* *: *p*-value of spearman’s rho correlation coefficients between uremic toxins and residual urine volume (expressed in mL/24 h/m^2^). Significant *p*-values are marked in bolt.
